# On the Drive Specificity of Freudian Drives for the Generation of SEEKING Activities: The Importance of the Underestimated Imperative Motor Factor

**DOI:** 10.3389/fpsyg.2018.00616

**Published:** 2018-05-03

**Authors:** Michael Kirsch, Wolfgang Mertens

**Affiliations:** ^1^Institute of Physiological Chemistry, University Hospital Essen, Essen, Germany; ^2^Division of Clinical Psychology and Psychotherapy, Department of Psychology, Faculty of Psychology and Educational Sciences, Ludwig Maximilian University of Munich, Munich, Germany

**Keywords:** ghrelin, angiotensin II, testosterone, adenosine, drive specificity

## Abstract

Doubters of Freud’s theory of drives frequently mentioned that his approach is outdated and therefore cannot be useful for solving current problems in patients with mental disorders. At present, many scientists believe that affects rather than drives are of utmost importance for the emotional life and the theoretical framework of affective neuroscience, developed by Panksepp, strongly underpinned this view. Panksepp evaluated seven so-called command systems and the SEEKING system is therein of central importance. Panksepp used Pankseppian drives as inputs for the SEEKING system but noted the missing explanation of drive-specific generation of SEEKING activities in his description. Drive specificity requires dual action of the drive: the activation of a drive-specific brain area and the release of the neurotransmitter dopamine. Noticeably, as Freud claimed drive specificity too, it was here analyzed whether a Freudian drive can evoke the generation of drive-specific SEEKING activities. Special importance was addressed to the imperative motor factor in Freud’s drive theory because Panksepp’s formulations focused on neural pathways without specifying underlying neurotransmitter/endocrine factors impelling motor activity. As Panksepp claimed sleep as a Pankseppian drive, we firstly had to classified sleep as a Freudian drive by using three evaluated criteria for a Freudian drive. After that it was possible to identify the imperative motor factors of hunger, thirst, sex, and sleep. Most importantly, all of these imperative motor factors can both activate a drive-specific brain area and release dopamine from dopaminergic neurons, i.e., they can achieve the so-called drive specificity. Surprisingly, an impaired Freudian drive can alter via endocrinological pathways the concentration of the imperative motor factor of a second Freudian drive, obviously in some independence to the level of the metabolic deficit, thereby offering the possibility to modulate the generation of SEEKING activities of this second Freudian drive. This novel possibility might help to refine the general understanding of the action of Freudian drives. As only imperative motor factors of Freudian drives can guarantee drive specificity for the generation of SEEKING activities, the impact of Freud’s construct *Eros* (with its constituents hunger, thirst, sex, and sleep) should be revisited.

## Introduction

In psychoanalysis, dealing with the motivational reasons for an action was, and is, of central significance. For many years,Freud’s “theory of drives” – he himself spoke of “Trieblehre^1^” (Freud, 1905a,b, 1914a,b, 1920b) – endeavored to do this. In the final version, Freud (1940, p. 71) drew a distinction between the “*Eros*,” whose goal was the production of larger and larger units, and the “destructive drive” or “death drive” (often called “*Thanatos*”), whose aim was the dissolution of contexts. In 1923, Freud clarified that two Freudian drives are constituent elements of *Eros*: “*According to this view we have distinguish two classes of instincts, one of which, the sexual instinct or Eros, is by far the more conspicuous and accessible to study. It comprises not merely the uninhibited sexual instinct proper and the instinctual impulses of an aim-inhibited or sublimated nature derived from it, but also the self-preservative instinct,…*” (Freud, 1923, p. 3974). In earlier works, he distinguished between the sexual instinct and the self-preservation ones, and between the ego libido (or narcissistic libido) and the object libido.

Freud’s main concern was to understand how the psychosexuality that emerges at the beginning of life unconsciously influences the ongoing development of an individual, his personality, his ability to love, interaction with other people, and his professional preferences and aversions. It does this in an infinite number of manifestations, of course not only in the form of genital pleasure or sexual dysfunction.

Some mistranslations of the German nouns “Drang” (correctly in the sense of Freud: imperative motor factor) as “motor factor,” “Trieb” (correctly: drive) as “instinct” and “Trieblehre” (correctly in the sense of Freud: theory of motivational drives) as “theory of instincts” have given rise to the misconception that drive is, in its origins, an exclusively biological occurrence which, following a certain triggering stimulus, always follows an identical pattern. In this way, the truly revolutionary aspects of the Freudian conception became lost, namely the psychological extension of the sex drive which was originally conceived of as being purely biological.

Psychoanalysis has been dealing with Freud’s concepts of drives/instincts for over a century. Ongoing controversial assessments currently prevail in the discussions about what is meant by an impulse, how many drives are to be assumed, how instincts and drives differ from each other, whether Freud’s views on libido and the death drive can be maintained, what motivational role these impulses play, what their influence is on the emergence of mental disorders, whether emotions precede the drives, whether infantile psychosexuality is already based on a biological event or is due to the intersubjective mediation of enigmatic messages on the part of adults^2^ (Laplanche, 2003).

From the perspective of many contemporary psychoanalysts, it is not drives which are the primary building blocks of emotional life, but affects. On the basis of more recent findings from the research on the phylogenesis and ontogenesis of affective systems (e.g., Ekman, 1982), it can now be assumed that affective signals already occur in the infant, sometimes even at the prenatal stage (Krause, 1983). Affects are not, as Freud supposed, manifestations of drives, but rather represent a relatively independent behavioral control system. Thus, sexuality is regarded not as a reduction in tension of accumulated libido, but rather as the desire for sexual activity is triggered by affects such as interest, longing, desire, or love.

The neuropsychologist Jaak Panksepp assumes that our consciousness does not come into being in higher, cortical centers, but already in subcortical regions of the brain, in innate, basal emotional systems (Panksepp, 1998). Up to now seven so-called command systems (labeled SEEKING, RAGE, FEAR, LUST, CARE, PANIC, and PLAY) have been identified by studying the rewarding and punishing effects during deep brain stimulations (Panksepp, 1998, 2016; Watt and Panksepp, 2009; Zellner et al., 2011; Solms and Panksepp, 2012; Wright and Panksepp, 2012; Panksepp and Yovell, 2014; Alcaro et al., 2017). The SEEKING system is of particular significance for the concept of *Eros* in this context because it refers to general positive motivations (Wright and Panksepp, 2012). SEEKING can be characterized by curiosity, exploration, and the longing for new meaningful experiences. It not only helps us cope with various requirements of life, but also aids the unremitting libidinal search for liveliness and self-realization.

In Figure 4 of the latter article (p. 17), Wright and Panksepp used (primary) homeostatic drives as inputs for the generation of SEEKING activities. Such a concept can only operate with confidence when each drive is able to specifically activate the (non-specific) SEEKING system, e.g., “*The ability to process and “decide” between the drives might be lost if each drive is not also an independent generator. In other words, we have to sustain drive-specificity even as they converge into a central processing unit such as the primary SEEKING emotion.*” (Wright and Panksepp, 2012, p. 18). Noticeably, drive specificity was also claimed by Freud: “*What distinguishes the instincts from one another and endows them with specific qualities is their relation to their somatic sources and to their aims*” (Freud, 1905b, p. 1492). On the thought that a Freudian drive, i.e., all constituents of *Eros*, can operate specifically in such a manner, we faced an unresolved problem. Although Freud was optimistic that the theory of motivational drives can be supported in principle by experimental data “*The deficiencies in our description would probably vanish if we were already in a position to replace the psychological terms by physiological or chemical ones.*” (Freud, 1920a, p. 54), his “Triebtheorie” has been never received any serious transfer of biochemical knowledge and because of this ignorance it is an outdated theoretical framework from a biochemical point of view.

The following advocates maintaining the *Eros* construct on the basis of new biochemical (and endocrinological) research output because only Freudian drives have in fact the kind of drive specificity for the generation of SEEKING activities that was requested by Panksepp.

## Sleep as a Freudian Drive

The thought to make the connection between Freudian drives and Panksepp’s SEEKING system required intrinsically to classify sleep as a Freudian drive because Wright and Panksepp (2012) stated: “*It may well be that an individual in the thrall of an overly focused SEEKING system becomes less likely/able to experience and bring about motivations related to even the most basic needs of an organism–that is, those related to the instinctual drives, ranging from hunger to sleep*” (p. 26). From Freud’s own notations “*A better term for an instinctual stimulus is a ‘need’.*” (Freud, 1915a, p. 118) and “*This view of the matter is supported by the fact that merely being awake, without doing any work, gives rise to fatigue and produces a need for sleep.*” (Breuer and Freud, 1895, p. 172) it can be assumed that the proclamation of sleep as a drive was even somewhat foreshadowed by him, albeit not mentioned explicitly.

In order to classify sleep with confidence as a Freudian drive, three Freudian drive criteria were discovered. Remarkably, the use of non-psychological parameters for identification of a Freudian drive was recommended by Freud himself: “*I am altogether doubtful whether any decisive pointers for the differentiation and classification of the instincts can be arrived at on the basis of working over the psychological material. This working-over seems rather itself to call for the application to the material of definite assumptions concerning instinctual life, and it would be a desirable thing if those assumptions could be taken from some other branch of knowledge and carried over to psychology.*” (Freud, 1915a, p. 124).

The advocated three Freudian drive criteria are:

(I) Imperative character.
  Freud himself noted that a drive [that “*does not arise from the external world*” (Freud, 1915a, p. 118)] in his sense is imperative: “*Let us take the case in which an instinctual stimulus such as hunger remains unsatisfied. It then becomes imperative and…*” (Freud, 1915b, p. 2977).
(II) Characteristic central orchestration by the lateral hypothalamus.
  Since an electrical stimulation of the lateral hypothalamus can evoke feeding (Delgado and Anand, 1953), drinking (Mogenson and Stevenson, 1967), or sexual behavior (Vaughan and Fisher, 1962), it is predicted that this brain area orchestrate generally Freudian drives.
(III) Characteristic central termination of Freudian drives by 5-hydroxytryptamine.

Freud described precisely the termination of the sexual drive: “*The new sexual aim in men consists in the discharge of the sexual products. The earlier one, the attainment of pleasure, is by no means alien to it; on the contrary, the highest degree of pleasure is attached to this final act of the sexual process*” (Freud, 1905b, p. 1524). Two research groups reported independently that the neurotransmitter 5-hydroxytryptamine is released in the brain during ejaculation in male rats (Mas et al., 1987, 1995; Lorrain et al., 1997; Hull et al., 2004). In addition, a pharmacologically mediated increase of the neurotransmitter 5-hydroxytryptamine can cause sexual dysfunctions in men (decreased sexual desire, erectile difficulties, and delayed ejaculation; Waldinger and Oliver, 1998).

The sexual-drive-dependent release of 5-hydroxytryptamine in women is experimentally harder to demonstrate because the hormone estrogen is suspected to temporally prolong the intermediacy of this neurotransmitter (Uphouse and Guptarak, 2010). However, in human beings, the 5-hydroxytryptamine-mediated sexual neurotransmission did not obviously depend on gender, because erectile dysfunctions as well as low vaginal lubrications are common side effects for patients with pharmacologically increased central 5-hydroxytryptamine levels (Goldstein and Goodnick, 1998; Rosen et al., 1999).

The neurotransmitter 5-hydroxytryptamine is also centrally released during the satisfaction of hunger. For instant, the eating of a palatable meal (a mash of chow and condensed milk) enhances the 5-hydroxytryptamine levels in samples of the lateral and the medial hypothalamus of normal rats (Schwartz et al., 1989, 1990; Mori et al., 1999). In line with these findings, application of a glucose solution (infusion or ingestion) elevates cerebral levels of 5-hydroxytryptamine (Vahabzadeh et al., 1995; Yamauchi et al., 1995). In addition, a pharmacological mediated increase of the cerebral 5-hydroxytryptamine level reduces food intake and body weight in animals (Nielsen et al., 1992) and, most informatively, also in humans (Ward et al., 1999).

By using these three Freudian drive criteria sleep is suggested here as a Freudian drive.

(I) The imperative nature of sleep is beyond dispute.
(II) Sleep is orchestrated by the lateral hypothalamus (Saper et al., 2010; Brown et al., 2012; Gutierrez Herrera et al., 2017).
(III) Although 5-hydroxytryptamine might be required for either the induction of sleep (Jouvet, 1969; Portas et al., 2000) or the promotion of it (Alenina et al., 2009), serotonin producing dorsal raphe nucleus neurons in cats are most active during waking (McGinty and Harper, 1976). It was further demonstrated in experiments with rats that the firing characteristics of dorsal raphe nucleus neurons during sleep correlates in fact with the production of 5-hydroxytryptamine and its concentration was therefore maximal during waking hours (Portas et al., 1998; Bjorvatn et al., 2002). This finding is confirmed by compounds that mimic the action of 5-hydroxytryptamine, i.e., so-called serotonin receptor agonists, because they all promote wakefulness (Monti, 2010). Thus, 5-hydroxytryptamine is a termination signal of sleep at the neurotransmitter level.

Now the question arises whether sleep, that cannot be classified as a component drive of the sexual ones, can be classified as a component drive of the self-preservative ones or (the possibility that we prefer) as a third constituents of *Eros*. Although some metabolites are regenerated (e.g., adenosine triphosphate is restored during sleep in wake-active brain regions and anabolic pathways are temporally stimulated; Hardie, 2007; Dworak et al., 2010) in a manner somewhat typical for the self-preservation drives (hunger and glucose vide infra), sleep has additional essential functions for the brain like maturation, cognitive processing, and sleep was found to enhance overnight memory consolidation (Perogamvros and Schwartz, 2012; Gutierrez Herrera et al., 2017). Because of these additional key functions, we believe that sleep cannot be assumed with confidence as a component drive of the (mainly metabolic imbalances restoring) self-preservation ones.

In any case, sleep can be safely assumed as a Freudian drive and this conclusion allows now the thought that Freudian drives in general may have some properties to generate SEEKING activities.

## Generation of Seeking Activities by the Hunger Drive

The properties of the hunger drive to generate SEEKING activities was analyzed by Wright and Panksepp (2012, pp. 16–17). At first, a Pankseppian drive was introduced “*Fortunately, the concept of “drive”, at least in neuroscience/physiology, has a more specific meaning-namely, states of imbalance in various bodily regulations, instantiated in such processes as hunger and thirst, which reflect actual activity of particular subcortical, especially hypothalamic, neural processes (Panksepp, 1981)*” (Wright and Panksepp, 2012, p. 16). Compared to the architecture of a Freudian drive with its known elements (i.e., somatic source, aim, object, imperative motor factor), a Pankseppian drive has only one element, i.e., the regulatory imbalance. The function of a Pankseppian drive was also clearly stated: “*Thus, each bodily drive has a distinct neural distribution with specific homeostatic regulatory functions, which exert some control over SEEKING urges (Figure 4).*” (Wright and Panksepp, 2012, p. 16). Therefore, the Pankseppian drive has obviously two quite different missions. At first, to stimulate a drive-specific brain area (in the case of hunger the arcuate nucleus; Wright and Panksepp, 2012, p. 17). At second, as “*the SEEKING system has been most closely associated with dopamine release*” (Wright and Panksepp, 2012, p. 11), the drive has to initiate the release of this neurotransmitter from dopaminergic neurons present in brain areas of the SEEKING system (i.e., ventral tegmental area, medial forebrain bundle, lateral hypothalamus, nucleus accumbens, and medial prefrontal cortex; Panksepp and Biven, 2012, p. 104). Again, the dual functionality of a drive was also claimed by Freud: “*There is a further provisional assumption that we cannot escape in the theory of instincts. It is to the effect that excitations of two kinds arise from the somatic organs, based upon differences of a chemical nature.*” (Freud, 1905b, p. 1492). Unfortunately, the biochemical explanations (i.e., the intermediacy of cholecystokinin, leptin, and neuropeptide-Y) given by Wright and Panksepp (2012) cannot clarify how these two brain areas were activated by the Pankseppian drive. The application of either cholecystokinin or leptin in experimental animals induces satiety signals thereby reducing feeding (Date et al., 2005; Kanoski and Grill, 2015). Thus, both gastrointestinal hormones are termination signals of the hunger drive at the endocrinological level and, of course, termination regulators of the drive of interest cannot explain the initiation of that process. The mentioned intermediacy of neuropeptide-Y is useless too for the understanding of the activation of the two brain areas, because a neurotransmitter can be a down-stream product of the (Pankseppian) drive activity but it cannot be a constituent of the hunger drive. Such uncertainties in the concept will predict erroneous functions of the SEEKING system. In fact, it has recently mentioned that the SEEKING command system runs into difficulties by describing the situation of a hungry baby crying for food for the first time (Bazan and Detandt, 2013). Because of this misinterpretation, evoked by the missing details how the two brain areas were activated, it is concluded that a Pankseppian drive fails in explaining any drive-specific generation of SEEKING activities. In order to confirm Panksepp’s claim that two different brain areas need to be activated to maintain drive specificity, a drive concept is necessary that included additionally a “molecular minister” of the metabolic imbalance.

A Freudian drive has in regard to a Pankseppian drive the conceptual advantage of an imperative motor factor, i.e., the molecular minister of the metabolic imbalance, that should be able to activate the two suggested brain areas. In order to clarify the lacking information, the hunger drive will now be analyzed in some detail by looking through the glasses of Freud’s drive theory.

It is known that a healthy human being becomes hungry when the blood glucose level falls below 8 mM (Andrews et al., 1998). Because of this decrease, it is possible to define: (somatic source = decrease of glucose, aim = increase of glucose, object = food). Neurobiologist have often noted that hunger (and appetite) is regulated via the hypothalamus by sensing both peripheral hormones (e.g., ghrelin) and key metabolites (e.g., glucose) (Messina et al., 2014; Gutierrez Herrera et al., 2017). The idea that the central nervous system senses glucose was introduced in 1849 (Bernard, 1849). Neurons in the hypothalamus have excitatory (and inhibitory) receptors for glucose and after an occupation of these targets the release of neurotransmitters, such as the neuropeptide orexin A, can be enhanced (or be suppressed) (Messina et al., 2014; Cone and Elmquist, 2016). Although this pathway is still an important one, the second possibility of mediating food intake via activation of the SEEKING reward-related system has attracted much attention, because incorporating psychological findings may help find rational strategies for combating obesity. The peripheral hormone ghrelin is mainly released by the stomach (Kojima et al., 1999; Stievenard et al., 2017) and its plasma concentration increases when the glucose level decreases (Klok et al., 2007; Vatansever-Ozen et al., 2011). It should be noted that the brain cannot synthesize ghrelin (Furness et al., 2011; Francois et al., 2015), and that therefore ghrelin cannot be classified as a neurotransmitter. In such a situation, the concentration of the termination signal at the neurotransmitter level, i.e., 5-hydroxytryptamine, also decreases (Donovan and Tecott, 2013). Targets for ghrelin were located in the ventral hippocampus (Kanoski and Grill, 2015), in the substantia nigra pars compacta, in the ventral tegmental area and, additionally, in the arcuate nucleus (Zigman and Elmquist, 2003; Zigman et al., 2006; Andrews et al., 2009; Lutter and Nestler, 2009; Skibicka et al., 2011; Mani et al., 2014; Wellman and Abizaid, 2015). Thus, ghrelin is the imperative motor factor of the Freudian hunger drive. Most informatively, an intraperitoneal injection of ghrelin in mice increases striatal dopamine levels (Stievenard et al., 2017). In fact, several manuscripts report about a ghrelin receptor (i.e., the target for ghrelin) on dopaminergic neurons in the ventral tegmental area and that an occupation of this receptor by ghrelin induced the release of dopamine (Naleid et al., 2005; Abizaid et al., 2006; Jerlhag et al., 2007). In the arcuate nucleus targets for ghrelin are found on neuropeptide-Y releasing neurons as well as on agouti-related peptide releasing ones and occupation of these receptors by ghrelin stimulate these neurons (Zigman and Elmquist, 2003; Lutter and Nestler, 2009; Stemson, 2013). Noticeably, neuropeptide-Y as well as agouti-related peptide increase feeding when injected into the brain (Clark et al., 1984; Levine and Morley, 1984; Ollmann et al., 1997). Most impressively, mice with an ablation of these neurons reject to consume food when it is even placed in their mouth (Wu et al., 2008). The essential character of the ghrelin-dopamine pathway for food intake is evidenced by observations that dopamine-deficient mice normally die of starvation but restores feeding after application of dopamine in the striatum (Szczypka et al., 1999, 2001). Thus, the imperative motor factor of the Freudian drive hunger, i.e., the peripheral hormone ghrelin, does double duty as requested by Panksepp: it activates the drive-specific brain area, i.e., the arcuate nucleus, and it induces the release of dopamine in the ventral tegmental area. However, according to Freud’s prediction “*the nervous system is an apparatus which has the function of getting rid of the stimuli that reach it, or reducing them to the lowest possible level;*” (Freud, 1915a, p. 120) there must be mechanisms to shut down the drive. After food intake, the plasma concentration of ghrelin decreases (Donovan and Tecott, 2013), while that of glucose as well as of the peripheral hormone leptin increases (Vatansever-Ozen et al., 2011). Leptin enters the brain by using a blood brain barrier transport system (Kanoski and Grill, 2015). Both glucose (vide supra) and leptin lead to the release of 5-hydroxytryptamine because intraperitoneal injections of leptin can also increase hypothalamic 5-hydroxytryptamine turnover (Ribeiro et al., 2009). This increase of the termination signal is expected to down-regulate the release of dopamine and thus to decrease the SEEKING reward-related activity (Patkina and Lapin, 1976; Katz and Carroll, 1977; Miliaressis, 1977; van der Kooy et al., 1977; Lee and Kornetsky, 1998; Benaliouad et al., 2007; McBride, 2010). In addition, inhibitory receptors for leptin are present on dopaminergic neurons (Billes et al., 2012; Donovan and Tecott, 2013), and this should further depress the release rate of dopamine (and therefore the corresponding SEEKING reward-related activity). Noticeably, glucose and leptin do (like ghrelin) also double duty because they additionally inhibit the neurons of the arcuate nucleus (Lutter and Nestler, 2009; Stemson, 2013), i.e., the neurons of the hunger drive-specific area.

Conclusively, in the case of hunger the Freudian drive can induce generation of SEEKING activities because its imperative motor factor, i.e., the peripheral hormone ghrelin, can activate the two brain areas mentioned by Panksepp and achieves thereby the requested drive specificity.

## Generation of Seeking Activities by Other Freudian Drives

In order to be able to achieve drive specificity in a general manner, it must now be claimed that other Freudian drives have different imperative motor factors with different targets in the brain but will also promote the release of dopamine from dopaminergic neurons. Unfortunately, these drives are less well evaluated but there are enough puzzle parts available for identifying the picture (**Table 1**).

**Table 1:** Specificities of the various imperative motor factors.

*Eros* constituent	Imperative motor factor	Drive-specific brain area
Hunger	Ghrelin	Arcuate nucleus
Thirst	Angiotensin II	Subfornical organ, area postrema, and OVLT
Sex (male)	Testosterone	Medial preoptic area
Sleep (NREM)	Adenosine	Tuberomammillary nucleus


*OVLT, organum vasculosum of the lamina terminalis; NREM, non-rapid eye movement.*

Inspection of **Table 1** demonstrated that each constituent of *Eros* has its own imperative motor factor and its corresponding specific area in the brain. The astute reader may counter that other hormones or metabolites are equally important for Freudian drives thereby query the importance of our selection process. It should be noted that the suggested imperative motor factors do double duty in order to achieve drive specificity for the SEEKING system: they operate in the drive-specific brain area and they support the release of dopamine from dopaminergic neurons. For instance, systemically and centrally injected angiotensin II in rats induces thirst (Buggy and Johnson, 1978; Johnson and Thunhorst, 1997) and angiotensin II induces striatal dopamine release (Brown et al., 1996). The action of testosterone implants in castrated rats demonstrated that the hypothalamic-preoptic area is the causal factor in activation of sexual behavior (Davidson, 1966) and an intranasal concentration of testosterone in gonadally intact adult male rats increases both the concentration and the turnover of dopamine in the striatum (de Souza Silva et al., 2009; Losecaat Vermeer et al., 2016). Of course, the selection of the imperative motor factor for sleep is much more difficult because here are two phases [at first non-rapid eye movement (NREM) sleep and at second rapid eye movement sleep]. From Panksepp’s advice “*Other systems promote sleepiness, and in the midst of sleep we have dreams that are energized by dopamine-driven SEEKING urges*.” (Panksepp and Biven, 2012, p. 99) an imperative motor factor had to be found that on the one hand induces NREM sleep and on the other hand supports exclusively after the NREM period the release of cerebral dopamine. In fact, adenosine operating in the tuberomammillary nucleus promotes NREM sleep (Oishi et al., 2008) and adenosine receptor-mediated modulation of dopamine release in the nucleus accumbens depends on the presence of sufficient concentrations of the neurotransmitter glutamate (Quarta et al., 2004). This finding was quite important for our selection process because cerebral glutamate levels decreases during NREM sleep but increases during rapid eye movement sleep (Watson et al., 2010). Thus, as requested by Panksepp, the adenosine-dependent cerebral release of dopamine should only proceed in an effective manner during rapid eye movement sleep.

So it can confidently concluded that every constituent of *Eros* should be able to generate SEEKING activities because the Freudian drives exhibit the kind of drive specificity claimed by Panksepp. Provided that such a fusion between *Eros* and SEEKING can be accepted as an additional possibility, there is now a conceptual uncertainty that needs to be clarified by the scientific successors of Panksepp’s lifework, i.e., the claim of an external stimulus (Panksepp, 1998; Wright and Panksepp, 2012). When the external stimulus can be equated to the “object” of the Freudian drive, it would then be (as a part of the Freudian drive) a superfluous claim. Without such a revision we will face the dilemma that an internal stimulus is needed for the operation of the Freudian drive and a second external one is then required for the activation of the SEEKING system. Such a construct would be rejected by the majority of Freud followers.

## Key Importance of Imperative Motor Factors for the Generation of Seeking Activities

Panksepp believed that a metabolic imbalance (i.e., that corresponded to the somatic source in the Freud’s motivational drive theory) yields (in conjunction with the proposed external stimuli) directly to an activity of particular subcortical (hypothalamic) neural processes (vide supra; Panksepp, 1998; Wright and Panksepp, 2012). In contrast to this view, the imperative motor factors evoke obviously generation of SEEKING activities (vide supra). In order to discard any doubts, an additional example is offered. Caffeine is able to blockade the cerebral targets of the imperative motor factor adenosine (Watson et al., 2010). Thus, astute readers who are confident that coffee can shoo sleep away, intrinsically accepted the view that the imperative motor factor and not the level of the metabolic imbalance is connected with subcortical neural processes. In any case, two cups of morning coffee ingestion decreased sleep efficiency and overall sleep during the subsequent night (Landolt et al., 1995).

Because of this conclusion, an artificial disturbance of the imperative motor factor concentration would enhance (or depress) the generation of SEEKING activities in some independence to the corresponding level of its metabolic deficit. The arising question how such an alteration would occur can be answered: the concentration of the imperative motor factor of a Freudian drive-x can be artificially altered by a second (but impaired) Freudian drive-y. Of course, such an alteration via endocrinological pathways may be a down-streaming event (rather than a direct manipulation) of the impaired Freudian drive and two evaluated examples should highlight this hitherto ignored possibility.

### Example 1: An Impaired Sleep Drive Impairs the Hunger Drive

A persistent short habitual sleep time of around 5 h decreases leptin concentrations in blood by around 15% as well as increasing those of ghrelin by the same amount, and these alterations (increase of the imperative motor factor concentration and decrease of the drive termination signal concentration) lead to an increase in body mass index (Taheri et al., 2004). The view that the elevated ghrelin concentration is in fact responsible for the increase in body weight is somewhat supported by classical animal experiments because both intraperitoneal and intracerebroventricular administration of this imperative motor factor in non-hungry rats stimulated (obviously in independence to the level of the metabolic imbalance) an enhanced food intake for 24 h (Wren et al., 2000).

### Example 2: An Impaired Hunger Drive Impairs the Sexual Drive

There are very firm evidences that erectile dysfunction is more common among obese men than among men of normal weight (Kolotkin et al., 2012). Testosterone-deficient men have a decreased sexual activity (Kwan et al., 1983) and adipose tissue expresses the enzyme aromatase which converts testosterone to estradiol^3^ (Fui et al., 2014) thereby decreasing the concentration of the corresponding imperative motor factor.

In any case, the novel possibility that a change of the concentration of an imperative motor factor outside the brain by endocrinological processes (and in some independence to the level of the metabolic deficit) can modulate the generation of SEEKING activities may refine the general understanding of these processes.

## Conclusion

**FIGURE 1:**
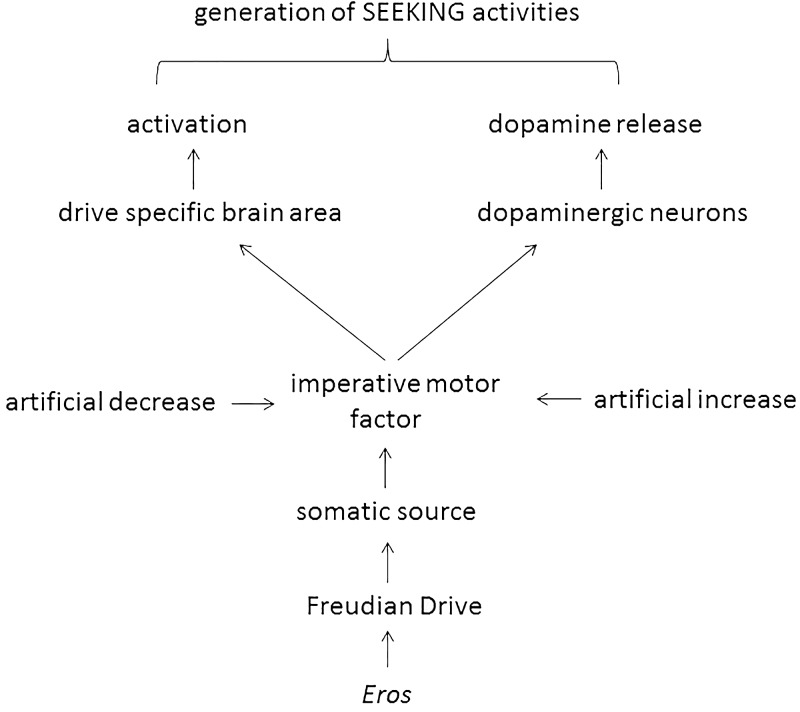
Scheme 1 | Proposed action of *Eros*-dependent generation of SEEKING activities.

This manuscript was written under the assumption that Freud’s 100-year-old theory of motivational drives is basically correct, albeit outdated. The only expansion – the introduction of sleep as a Freudian drive – has been done by respecting meticulously the instructions given by Freud. In the next step the imperative motor factor of various Freudian drives were evaluated at a biomolecular level (i.e., ghrelin, angiotensin II, testosterone, and adenosine; **Table 1**) and it was found that they all do double duty: they activate both a drive-specific area in the brain and dopaminergic neurons. Such an action considers a central claim of both Freud and Panksepp (i.e., drive specificity) and allows *Eros*-dependent generation of drive-specific SEEKING activities (**Figure 1**).

The consideration of actual findings on Freud’s “Triebtheorie,” which might be an update of it, leads to the following assertions:

1. Human beings are directed but not determined by Freudian drives in an unconscious manner.
2. The satisfaction of a Freudian drive leads to the release of the neurotransmitter 5-hydroxytryptamine in order to down-regulate the drive.
3. The sexual drive, hunger, thirst, and sleep are Freudian drives with an imperative character.
4. The imperative motor factor of a Freudian drive is a signal molecule that calls both the neurotransmitter dopamine and a second drive-specific neurochemical.
5. The imperative motor factor of a Freudian drive can evoke generation of drive-specific SEEKING activities.
6. The imperative motor factor of one Freudian drive can be modulated, in some independence to the level of its corresponding metabolic deficit, by another impaired constituent of *Eros*.

It has been noted in nearly countless amounts of manuscripts that Freud’s “Triebtheorie” will never be able to explain learning-dependent motivations such as compassion, empathy, altruism, and prosocial behavior in healthy human beings. Such a view might be somewhat revisited when an *Eros*-dependent generation of SEEKING [i.e., “*the “granddaddy” of all the emotional system.*” (Panksepp and Biven, 2012, p. 86)] activities would occur because this command system is also involved in learning processes (Kelley, 1999, 2004; Wright and Panksepp, 2012).

In summary, it can be said that the postulation of *Eros* within the context of classical Freudian motivational drive theory can still claim validity in accordance with contemporary psychoanalytic and multidisciplinary knowledge. At present, only the intermediacy of the imperative motor factor of the Freudian drives can explain convincingly a drive-specific generation of SEEKING activities.

## Author Contributions

MK had the original idea, introduced biochemical/endocrinologi-cal knowledge, wrote 75% of the text and revised the entire manuscript. WM introduced important psychological/psychanalytical knowledge, especially in relation to Freudian perspective and wrote 25% of the text.

## Conflict of Interest Statement

The authors declare that the research was conducted in the absence of any commercial or financial relationships that could be construed as a potential conflict of interest.

## Footnotes

1 Even with the “best” translation for “Trieblehre,” i.e., “theory of drives,” is its sense somewhat lost.

2 The questions raised at the outset are too complex to be answered in this paper.

3 This very helpful suggestion was advocated by a reviewer.
